# Lower Patellofemoral Joint Contact Force During Side-Step Cutting After Return-to-Sports Clearance Following Anterior Cruciate Ligament Reconstruction

**DOI:** 10.1177/03635465231166104

**Published:** 2023-05-15

**Authors:** Argell J. San Jose, Nirav Maniar, Rodney Whiteley, David A. Opar, Ryan G. Timmins, Roula Kotsifaki

**Affiliations:** * School of Behavioural and Health Sciences, Australian Catholic University, Fitzroy, Melbourne, Victoria, Australia; † OrthoSport Victoria Institute (OSVi), Richmond, Victoria, Australia; ‡ Sports Performance, Recovery, Injury and New Technologies (SPRINT) Research Centre, Australian Catholic University, Fitzroy, Victoria, Australia; § Department of Rehabilitation, Aspetar Orthopaedic and Sports Medicine Hospital, Doha, Qatar; ‖ School of Human Movement & Nutrition Sciences, The University of Queensland, Brisbane, Australia; # Oslo Sports Trauma Research Center, Department of Sports Medicine, Norwegian School of Sport Sciences, Oslo, Norway; Investigation performed at Aspetar Orthopaedic and Sports Medicine Hospital, Doha, Qatar

**Keywords:** ACL, biomechanics, knee, osteoarthritis

## Abstract

**Background:**

Low patellofemoral joint (PFJ) contact force has been associated with PFJ osteoarthritis. Quadriceps force and knee flexion angles, which are typically altered after an anterior cruciate ligament reconstruction (ACLR), primarily influence PFJ contact forces. It is still inconclusive whether differences in PFJ contact forces are present during high knee flexion tasks such as side-step cutting after clearance to return to sports (RTS) after ACLR.

**Purpose:**

To explore PFJ contact forces in the ACLR limb and compare them with those of the contralateral and control limbs during side-step cutting tasks after clearance to RTS.

**Study Design:**

Controlled laboratory study.

**Methods:**

A total of 26 male athletes with ACLR who were previously cleared to RTS were matched with 23 healthy men serving as the control group. Three-dimensional motion capture and force plate data were collected while both groups performed anticipated side-step cutting tasks. Joint kinematics, kinetics, muscle forces, and PFJ contact forces were calculated using musculoskeletal modeling.

**Results:**

Peak PFJ force was lower in the ACLR limbs compared with the contralateral limbs (mean difference [MD], 5.89 body weight [BW]; 95% CI, 4.7-7.1 BW; *P* < .001) and the control limbs (MD, 4.44 BW; 95% CI, 2.1-6.8 BW; *P* < .001). During peak PFJ force, knee flexion angle was lower in ACLR limbs compared with the contralateral (MD, 4.88°; 95% CI, 3.0°-6.7°; *P* < .001) and control (MD, 6.01°; 95% CI, 2.0°-10.0°; *P* < .002) limbs. A lower quadriceps force compared with the contralateral (MD, 4.14 BW; 95% CI, 3.4-4.9 BW; *P* < .001) and control (MD, 2.83 BW; 95% CI, 1.4-4.3 BW; *P* < .001) limbs was also found.

**Conclusion:**

Lower PFJ contact forces and a combination of quadriceps force deficits and smaller knee flexion angle were found in the ACLR compared with the contralateral and control limbs even after clearance to RTS.

**Clinical Relevance:**

Despite rehabilitation and subsequent clearance to RTS, differences in PFJ contact forces are present after ACLR. Current rehabilitation and RTS battery may not be effective and sensitive enough to identify and address these differences.

Rupture of the anterior cruciate ligament (ACL) is one of the most common injuries in the knee.^39^ Typical management of an ACL rupture usually includes ACL reconstruction (ACLR)^52^ followed by ~6 to 12 months of rehabilitation, with the goal of restoring knee joint stability.^17^ Despite this, poor patient-reported outcomes related to knee function,^18^ high reinjury risk,^1^ and accelerated onset of knee osteoarthritis^28^ are common after ACLR. The development of knee osteoarthritis has been reported as early as 3 years after ACLR.^28^ Given the high rates of ACLR in young athletes (<25 years),^39^ early knee joint degeneration can lead to a significant number of young individuals with impaired function and reduced quality of life because of knee osteoarthritis.^18^

Alterations in lower limb biomechanics are common after ACLR.^22^ Smaller knee flexion angle and excursion as well as lesser knee extension moments are commonly reported in the ACLR leg compared with the contralateral leg and healthy controls during tasks like gait and running.^22,30^ Furthermore, lower knee joint contact forces are common after ACLR,^31,53^ with lower knee joint contact force during walking associated with the development of knee osteoarthritis 5 years after ACLR.^53^ Most of the studies on knee joint contact force and osteoarthritis risk after ACLR have focused on the tibiofemoral joint.^20,43,53^ However, patellofemoral joint (PFJ) osteoarthritis is reported to be as high as 80% after ACLR^26^ and is associated with worse disabilities compared with osteoarthritis in other knee compartments.^12^ Therefore, identifying possible mechanisms related to the increased risk of PFJ osteoarthritis may be important to improve patient outcomes after ACLR.

Throughout ACLR rehabilitation, individuals progress from normal gait tasks to more dynamic movements such as running, jumping, and side-step cutting.^17^ Of these tasks, the side-step cut is one of the most physically demanding and commonly performed tasks in team sports and is a common mechanism of ACL injury.^10^ During side-step cutting, large loads in the PFJ can occur because of the high knee flexion angles and quadriceps force commonly seen during the execution of the task.^7^ The interaction between knee flexion angle and quadriceps force determines the total compressive forces at the PFJ.^23^ Given that quadriceps weakness^6^ and reduced knee flexion angle during tasks (eg, side-step cutting)^43^ are common in individuals who have undergone ACLR, these could potentially lead to alterations in PFJ contact forces. Previous studies have investigated PFJ contact forces after ACLR during walking, running, and single-leg forward hopping.^4,24,45,46,54^ The results from these studies suggest that reductions in PFJ contact forces could be secondary to the presence of reduced quadriceps strength, lower peak knee flexion angles, or both, as well as psychological factors related to fear of reinjury and/or instability and compensatory strategies to underload the ACLR limb. However, no study has yet investigated PFJ contact force in individuals who have undergone ACLR and have successfully passed return-to-sports (RTS) criteria (eg, quadriceps strength symmetry >90%). Furthermore, PFJ contact force during a high-demand task like side-step cutting has yet to be examined.

Therefore, the aim of this study was to investigate PFJ contact forces during the performance of a side-step cutting task at the time of RTS clearance in individuals who have undergone ACLR and compare them with those of the contralateral limb and a healthy control group. Our hypothesis was that there would be lower PFJ contact forces in the ACLR limb compared with the contralateral and healthy control limbs.

## Methods

### Participants

**Figure 1.**
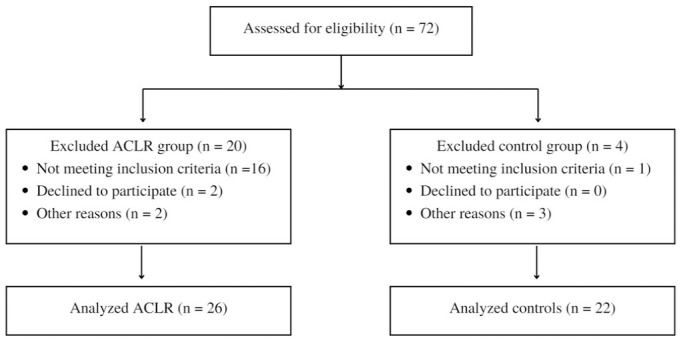
Study flow diagram. ACLR, anterior cruciate ligament reconstruction.

A total of 48 participants agreed to take part in this study (Figure 1): 26 men who had been cleared for RTS after ACLR and 22 healthy men who served as the control group. Participants in the ACLR group were athletes (preinjury Tegner score ≥7) between 18 and 35 years old who had a unilateral ACLR using either a hamstring tendon–semitendinosus + gracilis (n = 10) or a bone–patellar tendon–bone (n = 16) autograft.

The ACLR group was recruited for the study after completing supervised rehabilitation at the Aspetar Orthopaedic and Sports Medicine Hospital and were subsequently enrolled 1 week after receiving RTS clearance. Clearance to RTS was conducted using a shared decision-making strategy^15^ that included consideration of the following: (1) clearance from both their surgeon and physical therapist, (2) completion of a sports-specific on-field rehabilitation program, (3) quadriceps strength (limb symmetry index ≥90%), and (4) hop test battery performance (limb symmetry index ≥90%).^33^ Participants with concomitant meniscal injury that did not significantly interfere with their rehabilitation were included in the study. Exclusion criteria for the study included full-thickness articular cartilage lesion and any other major lower extremity injury in both legs (eg, concomitant grade 3 knee ligament injury other than ACL). The activity levels for the ACLR (before ACL injury) and control groups were assessed using the Tegner Activity Scale.^5^ Patient-reported outcomes related to pain, function, and psychological readiness were collected using the International Knee Documentation Committee^27^ and Anterior Cruciate Ligament–Return to Sport after Injury^51^ questionnaires.

Recruitment for the control group was performed using a convenience sampling from a pool of professional and high-level recreational athletes. Inclusion criteria were age between 18 and 35 years, Tegner score ≥7, participation in level 1 or 2 sports (≥3 times per week), no previous lower limb surgery, and no lower limb muscle injury in the 3 months before testing.

### Study Design

Data collection for this controlled laboratory study was performed at the biomechanics laboratory of the Aspetar Orthopaedic and Sports Medicine Hospital. Participants were recruited between November 2018 and October 2019. Informed consent was obtained from all participants before participating in the study. This study is part of a larger study investigating RTS criteria after ACLR rehabilitation.^32^

Ethics approval for this study was granted (institutional review board F2017000227). The transfer and use of previously collected and nonidentifiable data was approved by the Australian Catholic University Human Research Ethics Committee (registration No. 2021-29N).

### Data Collection and Instrumentation

A total of 43 reflective markers were placed according to a full-body Plug-in Gait marker set that included additional anatomic markers on the sacrum, medial knee, and medial ankle.^13^ Three marker clusters were also placed laterally on the thigh and shank of both legs.^16^ Three-dimensional marker trajectories were collected using a 14-camera motion capture system (250 Hz; Vicon) along with ground-reaction forces using 5 ground-embedded force plates (1000 Hz; Kistler).

All participants wore shorts and shoes for data collection. Participants were familiarized with all procedures and tasks before data collection. Before biomechanical testing, participants performed a 7-minute warm-up session composed of running, side running, deep squats, and double-leg jumps.

For the side-step cut task, participants started in a standing position 6 m away from the force plates. They were then instructed to accelerate maximally toward the force plates, performing 3 trials of an anticipated 45° side-step cut to the left and to the right. The order of testing for each limb was randomized using a coin toss. For all tests, a clear foot contact of the plant foot (side-step cutting) on the force plate was needed for a trial to be considered successful.

### Musculoskeletal Modeling

Data analysis was performed using previous methods as described by Maniar et al,^37,38^ which include semiautomated analysis via a custom R code (R Core Team, 2020) interface with OpenSim Version 4.2.^14^ A generic musculoskeletal model was scaled to each individual's anthropometry based on a static trial.^34^ An inverse kinematics algorithm was used to calculate joint angles during the side-step cut by means of a weighted least squares optimization that minimizes the difference between model and experimental marker positions during the dynamic trials.^36^ Inverse dynamics was used to obtain the generalized forces and moments that are responsible for these movements. Static optimization was used to decompose joint moments into individual lower limb muscle forces by minimizing a cost function (sum of muscle activations squared). To calculate PFJ contact force, we used a separate empirically based model as described previously^19^:

$$F_{PFJ}=\sqrt{F_{Q}^{2}+F_{P}^{2}+2F_{Q}F_{P}\cos\beta },$$.

where *F_PFJ_* is the PFJ contact force, *F_Q_* is the quadriceps force, *F_P_* is the patellar tendon force, and *β* is the patellar mechanism angle. Note that *F_P_* and *β* were calculated as a function of the knee flexion angle and quadriceps force (calculated from the model), based on data from an in vitro study.^8^

### Data Analysis

The peak PFJ contact force was extracted during the stance phase (defined as the raw ground-reaction force exceeding 20 N). Because the primary determinants of the PFJ force are the knee flexion angle and quadriceps force,^19^ we also calculated the knee flexion angle and quadriceps force at the time of peak PFJ contact force.

### Validation and Verification

Qualitative verification of the temporal-varying characteristics of experimental joint angles (see Appendix Figure A1, available in the online version of this article) and moments (see Appendix Figure A2, available online) was comparable with that of previous work on side-step cutting in healthy individuals.^38^ The temporal characteristics between predicted muscle forces and electromyography (EMG) data from previous work performed^41^ throughout the stance phase of the side-step cut (see Appendix Figure A3, available online) also showed general agreement between model-based predictions and EMG data for most muscles, after accounting for EMG-to-force physiological delays (~100 ms) as per recommendations.^25^

### Statistical Analysis

Descriptive statistics were used to summarize participant characteristics. The Shapiro-Wilk test was used to check for normality of distribution of data.^44^ An independent-samples *t* test was used (*P*≤ .05) to determine between-group comparisons in participant characteristics. A linear mixed-effects model^2^ approach was used to determine if differences existed between the ACLR leg and the contralateral leg as well as the healthy control legs for each of the previously described outcome variables. For each linear model, the leg (ACLR, contralateral, healthy control) was modeled as a fixed effect and the participant number modeled as a random effect, while adjusting for approach velocity (ie, average center-of-mass forward velocity in the 50 ms before foot contact). Approach velocity was adjusted for, as any variation between groups or trials (eg, participants may run slower when cutting on their ACLR leg compared with healthy-leg cuts) could confound analysis if unaccounted for. Where significant effects were found for the limb, we conducted post hoc pairwise comparisons using the Tukey method.^35^ Data assumptions (eg, distributions) were verified via the visual inspection of qqplots and residual plots. For all analysis, statistical significance was set at *P* < .05 (statistical software: RStudio: Integrated Development for R. RStudio, Boston).Results

## Results

**Figure 2.**
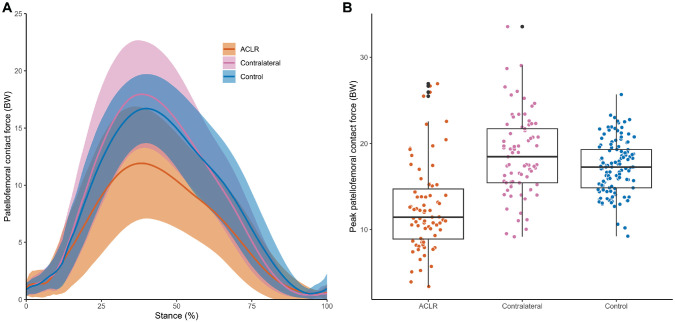
(A) Patellofemoral joint contact force during the stance phase of a side-step cut. Solid line and shaded area represent the mean and SD of the patellofemoral contact force across the stance phase, respectively. (B) Peak patellofemoral joint contact force between the anterior cruciate ligament reconstruction (ACLR), contralateral, and control limbs during the stance phase of a side-step cut. Dots represent all trials, the horizontal line inside the box represents the median, the edge of the boxes are the first and third quartiles, and vertical line represents the range of the peak patellofemoral joint contact force among the 3 groups. BW, body weight.

**Figure 3.**
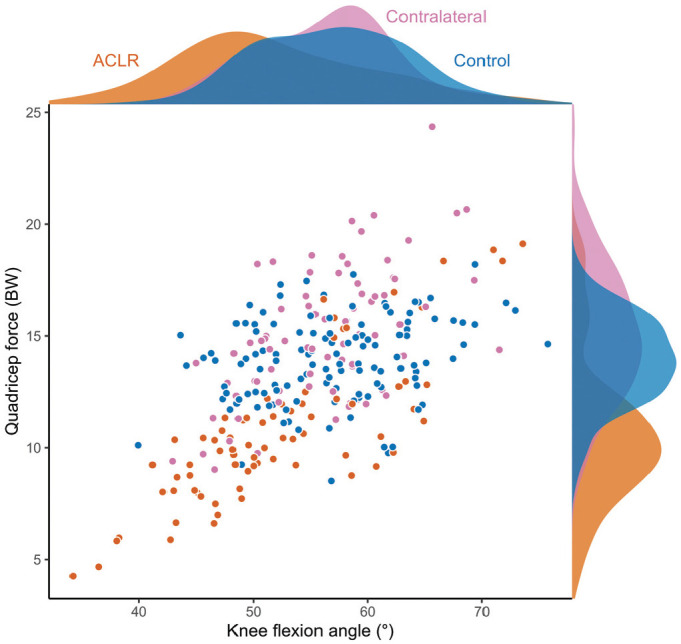
Knee flexion angle and quadriceps force at peak patellofemoral joint contact force for the anterior cruciate ligament reconstruction (ACLR), contralateral, and control limbs. The shaded region outside the box represents the probability density of the knee flexion angle (top) and quadriceps force (right) across the 3 groups. BW, body weight.

Patient characteristics and RTS testing results can be found in Table 1. The mean approach velocity for the side-step tasks was 3.7 ± 0.6 m/s for the ACLR leg, 3.9 ± 0.5 m/s for the contralateral leg, and 4.3 ± 0.6 m/s for the healthy control leg. The peak PFJ force was significantly less in the ACLR limbs compared with the contralateral limbs (mean difference [MD], 5.9 body weight [BW]; 95% CI, 4.7 to 7.3 BW; *P* < .001) (Figure 2, Table 2) and the control limbs (MD, 4.4 BW; 95% CI, 2.0 to 6.8 BW; *P* < .001) (Figure 2, Table 2). At the time of peak PFJ force, ACLR limbs had more extended knee joint angles compared with the contralateral (MD, 4.5°; 95% CI, 2.6° to 6.5°; *P* < .001) (Figure 3, Table 2) and control (MD, 5.7°; 95% CI, 1.6 to 9.8; *P* < .004) limbs (Figure 3, Table 2), as well as lower quadriceps force compared with the contralateral (MD, 4.3 BW; 95% CI, 3.5 to 5.1 BW; *P* < .001) (Figure 3, Table 2) and control (MD, 2.83 BW; 95% CI, 1.3 to 4.4 BW; *P* < .001) limbs (Figure 3, Table 2). No significant differences between contralateral and control limbs were observed for peak PFJ force (MD, 1.45 BW; 95% CI, –0.8 to 3.7 BW; *P* = .281) (Figure 2, Table 2), knee flexion angle (MD, 1.12°; 95% CI, –2.8° to 5.0°; *P* = .768) (Figure 3, Table 2), or quadriceps force (MD, 1.31 BW; 95% CI, –0.1 to 2.7 BW; *P* = .080) (Figure 3, Table 2) at the time of peak PFJ force. The relationship between knee flexion angle and quadriceps force at the time of peak PFJ contact force qualitatively shows that ACLR limbs tend to have a combination of smaller knee flexion angle and lower quadriceps force at peak PFJ contact force compared with healthy limbs (Figure 3).

**Table 1:** Participant Characteristics, Patient-Reported Outcome Measures, Quadriceps Strength, and Single-Leg Hop for Distance Performance Used as Criteria for Clearance to RTS^*a*^

	ACLR Group (n = 26)	Control Group (n = 22)	*P* Value
Age, y	23.2 ± 3.4	28.3 ± 4.3	<.001
Body mass, kg	71.4 ± 12.1	76.4 ± 7.3	0.08
Height, cm	173 (166 to 182)	178 (174 to 182)	0.19
Body mass index	23.3 ± 2.3	24.1 ± 1.6	0.18
Tegner score preinjury	9 (9 to 9)	7 (7 to 8)	<.001
IKDC	94.9 ± 7.1	99.7 ± 0.6	0.002
ACL-RSI	92.0 ± 10.8	NA	NA
Quadriceps strength LSI, %	94 ± 6	NA	NA
SLHD LSI, %	97 ± 4	100 ± 5	0.02
TRHD LSI, %	97 ± 5	100 (98 to 102)	0.09
RTS, mo	9.5 ± 2.7	NA	NA
ACL hamstrings autograft, n (%)	10 (38)	NA	NA
Isolated ACL injury, n (%)	14 (54)	NA	NA
Meniscal injury, n (%)	12 (46)	NA	NA
Cartilage lesion, n (%)	2 (8)	NA	NA

a Data are presented as mean ± SD for normally distributed data and median (interquartile range) for nonnormally distributed data, unless otherwise stated. ACL, anterior cruciate ligament; ACLR, anterior cruciate ligament reconstruction; ACL-RSI, Anterior Cruciate Ligament–Return to Sport after Injury; IKDC, International Knee Documentation Committee Subjective Knee questionnaire; LSI, limb symmetry index; NA, not available; RTS, return to sports; SLHD, single-leg hop for distance; TRHD, triple hop for distance.

**Table 2:** Peak PFJ Contact Force, Knee Flexion Angle at Peak PFJ Contact Force, and Quadriceps Force at Peak PFJ Contact Force^*a*^

	Peak PFJ Contact Force, BW	Knee Flexion Angle, Deg	Quadriceps Force, BW
ACLR	12.7 (11.3-14.1)^*b*^	52 (49-54)^*b*^	10.9 (10.0-11.7)^*b*^
Contralateral	18.7 (17.4-20.0)	56 (54-58)	15.1 (14.3-16.0)
Control	17.1 (15.7-18.5)	57 (55-60)	13.7 (12.8-14.6)

a Data are presented as marginal means (95% CI), accounting for approach velocity during the side-step cut. ACLR, anterior cruciate ligament reconstruction; BW, body weight; PFJ, patellofemoral joint.

b Significant difference compared with contralateral and control limbs (*P* < .05).

## Discussion

The most important finding of the study was that there were lower PFJ contact forces in the ACLR leg during the stance phase of a side-step cut when compared with the contralateral and healthy control limbs. Additionally, smaller knee flexion angle and lower quadriceps force were found at the time of peak PFJ contact force in the ACLR leg compared with the contralateral and healthy control limbs.

The PFJ contact forces found in this study (13-19 BW) (Table 2) were larger than those of the previous studies on walking (1.1-1.6 BW), running (3.4-6.7 BW), and single-leg forward hopping (8.6-10.8 BW) after ACLR.^29^ As this was the first study to investigate PFJ contact forces during a side-step cut, a comparative data set on the magnitude of our PFJ contact forces is currently not available. However, the magnitude of forces found in this study was not surprising given the larger knee flexion and knee extension moments required to perform a side-step cut compared with the abovementioned tasks.^48^ Studies on other activities that require larger knee flexion angles, such as a squat, showed that PFJ contact forces can go up to 18 BW.^23^

Previous studies have investigated PFJ contact forces during walking, forward hopping, and running in ACLR limbs compared with non-ACLR limbs.^4,24,45,46,54^ Similar to our results, lower PFJ contact forces in the ACLR limb were found during the stance phase of walking (3-6 months after ACLR)^54^ and running (1-2 years after ACLR)^4,45^ as well as the landing phase of a single-leg forward hop (1-2 years after ACLR)^46^ compared with the contralateral^4,24,45,46,54^ and healthy control groups.^24,46^ In contrast, Herrington et al^24^ found larger PFJ contact forces during the stance phase of running (~7 months after ACLR), whereas Williams et al^54^ found no differences between limbs during walking at 2 years. The differences of our results from these 2 studies could be attributed to the different tasks and time after ACLR. Herrington et al performed their assessments much earlier in the post-ACLR phase compared with our study during running tasks, whereas those of Williams et al were from a less demanding task (walking) at 2 years after ACLR.

Low knee flexion angle and knee extension moment during different tasks are common after ACLR.^22,30,46^ One of the proposed explanations for this is the presence quadriceps weakness.^21^ The presence of low quadriceps strength could logically explain a subsequent reduction in the ability to produce a knee extension moment. As such, biomechanical compensations such as a smaller knee flexion angle, as seen in the current study, or a relative increase in the joint moments produced at the trunk, hip, and ankle can arise from a reduced knee extension moment.^32,45^ Another explanation to the “underloading” of the knee joint in this study could be from psychological factors like pain, fear of reinjury, or psychological readiness. Previous studies have shown associations with low psychological readiness or fear of reinjury with aberrant lower limb biomechanics in individuals who have undergone ACLR.^49,56^ The combination of deficits in these physical and psychological capacities could potentially explain the smaller knee flexion angle, knee extension moment, and quadriceps force that resulted in the low PFJ contact forces in the ACLR limb compared with the healthy limbs in this study. However, the participants in this study had a relatively symmetrical isokinetic quadriceps strength limb symmetry index (Table 1) as well as satisfactory subjective perception of knee function and readiness (Table 1). Researchers have proposed that compensatory strategies can develop during the earlier phases of rehabilitation to achieve task completion despite the presence of deficits in physical and/or psychological capacity.^29^ It could be that despite restoration of strength and return of confidence and comfort in the knee, these strategies are still persistent at the time of RTS.

Lower PFJ contact force in the ACLR limb during a side-step cut compared with in the contralateral and healthy limbs, despite RTS clearance, may have implications for the development of knee osteoarthritis. Lower contact forces in the tibiofemoral joint during walking, 6 months after ACLR, have been associated with radiographic signs of tibiofemoral joint osteoarthritis in the ACLR leg.^53^ Similarly, lower PFJ contact forces during forward hop tasks have been related to radiographic signs of PFJ osteoarthritis as early as 1 year after ACLR.^11^ The reduction in PFJ contact force may have consequences for the articular cartilage. The cyclic application and removal of joint contact force is necessary for cartilage health.^9^ As such, a reduction in PFJ contact force may alter the normal load cycling of the cartilage and trigger a series of mechanical and metabolic changes that eventually lead to cartilage deterioration and onset of osteoarthritis.^9,50^ However, the association between lower PFJ contact forces and the development of PFJ osteoarthritis is still inconclusive and needs further investigation.

In addition to the lower peak PFJ forces, the influence of knee flexion angle on PFJ load location should be considered, given the observed differences in knee flexion during cutting tasks. Although not a focus of the current research, understanding the interaction between the location and magnitude of loading in the PFJ during cutting movements may shed light on the development of PFJ osteoarthritis after ACLR. To date, prospective studies investigating the effect of lower PFJ contact forces on the development of PFJ osteoarthritis are lacking. Future prospective studies are needed to better understand cartilage response to PFJ loading and the onset of osteoarthritis after ACLR.

We acknowledge that there are limitations to the current study. First, our PFJ contact force model only considered the sagittal plane biomechanics of the patella. Although frontal and transverse plane loading could potentially influence PFJ contact forces, PFJ loading is largely sagittal plane dominant, and the results of our study were relatively comparable with available data.^23,46^ Regardless, future studies could increase the complexity of the model to account for other planes. Second, our study was cross-sectional in nature, and we were not able to determine the biomechanical changes after ACLR and rehabilitation. The lower PFJ contact forces, smaller knee flexion angles, and lower quadriceps forces found in this study may have been present before ACL injury. Third, the participants in this study had either hamstring tendon or patellar tendon graft. Even though graft type morbidity is commonly reported in muscle strength,^55^ previous studies on the effect of graft type on knee osteoarthritis outcomes (radiographic changes, pain, function, symptoms) have been mixed.^3,47^ Given this, future work that compares PFJ contact forces after different ACLR graft types may be warranted. Last, this study included a male-only population from a single site, which limits the generalizability of our results to women. Future work in the female population is still needed given the previously reported differences in lower limb strength and biomechanics between the sexes.^40,42^

## Conclusion

This study found that PFJ contact forces during a side-step cut were lower in the ACLR limb when compared with contralateral and control limbs, despite clearance of athletes to RTS. A combination of reduction in quadriceps force and smaller knee flexion angle was found in the ACLR limb compared with the contralateral and healthy control limbs. Current RTS criteria do not appear effective enough to restore biomechanical alterations in the lower limbs that may predispose individuals who have undergone ACLR to lower PFJ contact forces.

## Supplemental Material

sj-pdf-1-ajs-10.1177_03635465231166104 – Supplemental material for Lower Patellofemoral Joint Contact Force During Side-Step Cutting After Return-to-Sports Clearance Following Anterior Cruciate Ligament Reconstruction Click here for additional data file. Supplemental material, sj-pdf-1-ajs-10.1177_03635465231166104 for Lower Patellofemoral Joint Contact Force During Side-Step Cutting After Return-to-Sports Clearance Following Anterior Cruciate Ligament Reconstruction by Argell J. San Jose, Nirav Maniar, Rodney Whiteley, David A. Opar, Ryan G. Timmins and Roula Kotsifaki in The American Journal of Sports Medicine
