# Supplementary material for: Lower Patellofemoral Joint Contact Force During Side-Step Cutting After Return-to-Sports Clearance Following Anterior Cruciate Ligament Reconstruction
Source: Am J Sports Med. 2023 May 15;51(7):1777–84. doi: 10.1177/03635465231166104 (PMC10240639; doi:10.1177/03635465231166104)
Supplement: sj-pdf-1-ajs-10.1177_03635465231166104 – Supplemental material for Lower Patellofemoral Joint Contact Force During Side-Step Cutting After Return-to-Sports Clearance Following Anterior Cruciate Ligament Reconstruction [file sj-pdf-1-ajs-10.1177_03635465231166104.pdf]

Lower patellofemoral joint contact force during side-step cutting after return to sports clearance after anterior cruciate ligament reconstruction

APPENDIX

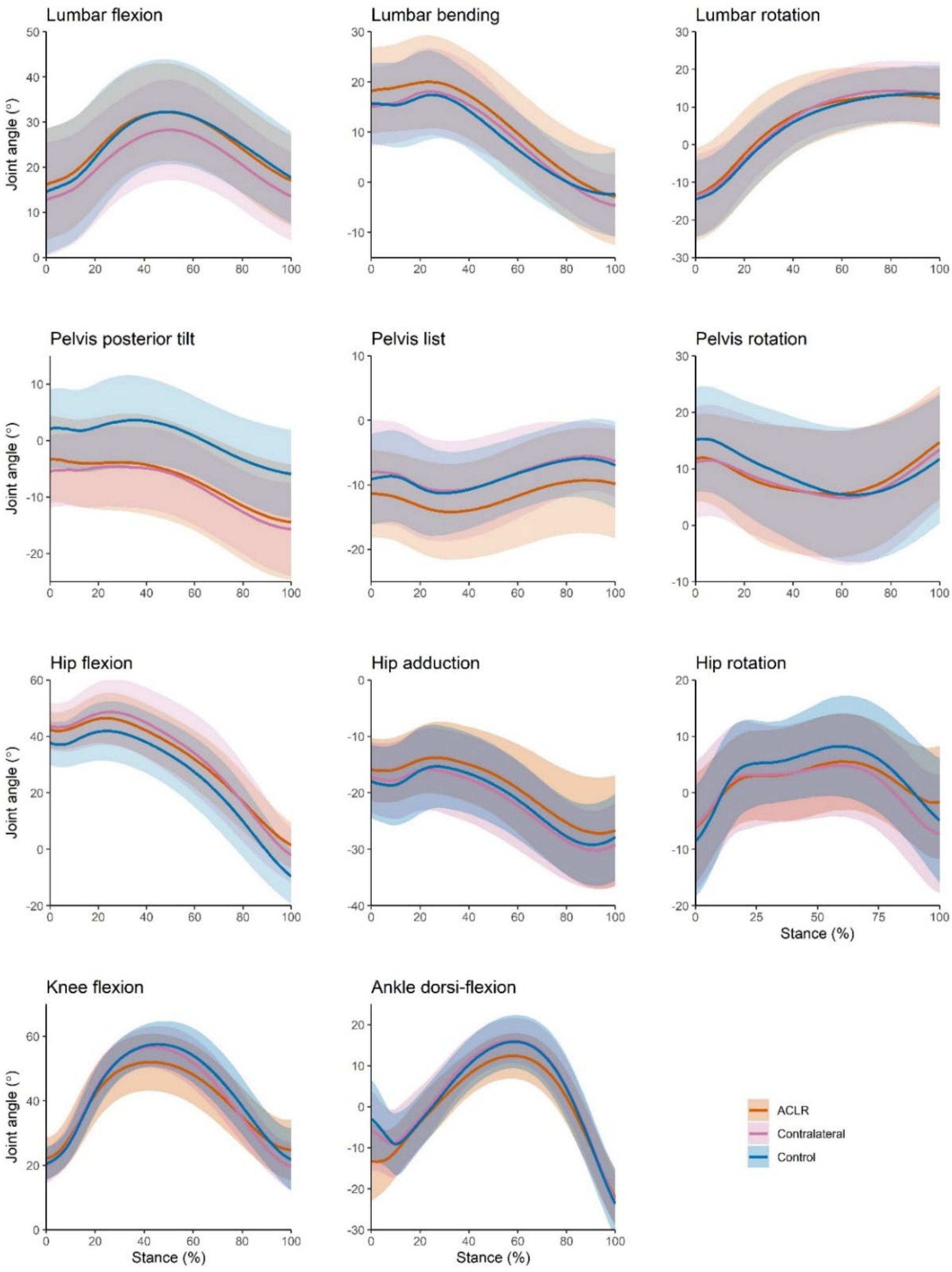

Figure A1. Mean (line) and SD (shaded area) joint angles during the stance phase of the anticipated sidestep cut between the ACLR (orange), Contralateral (pink), and Control (blue) limbs. Values are averaged across all participants and reported in degrees. *ACLR,* anterior cruciate ligament reconstruction.

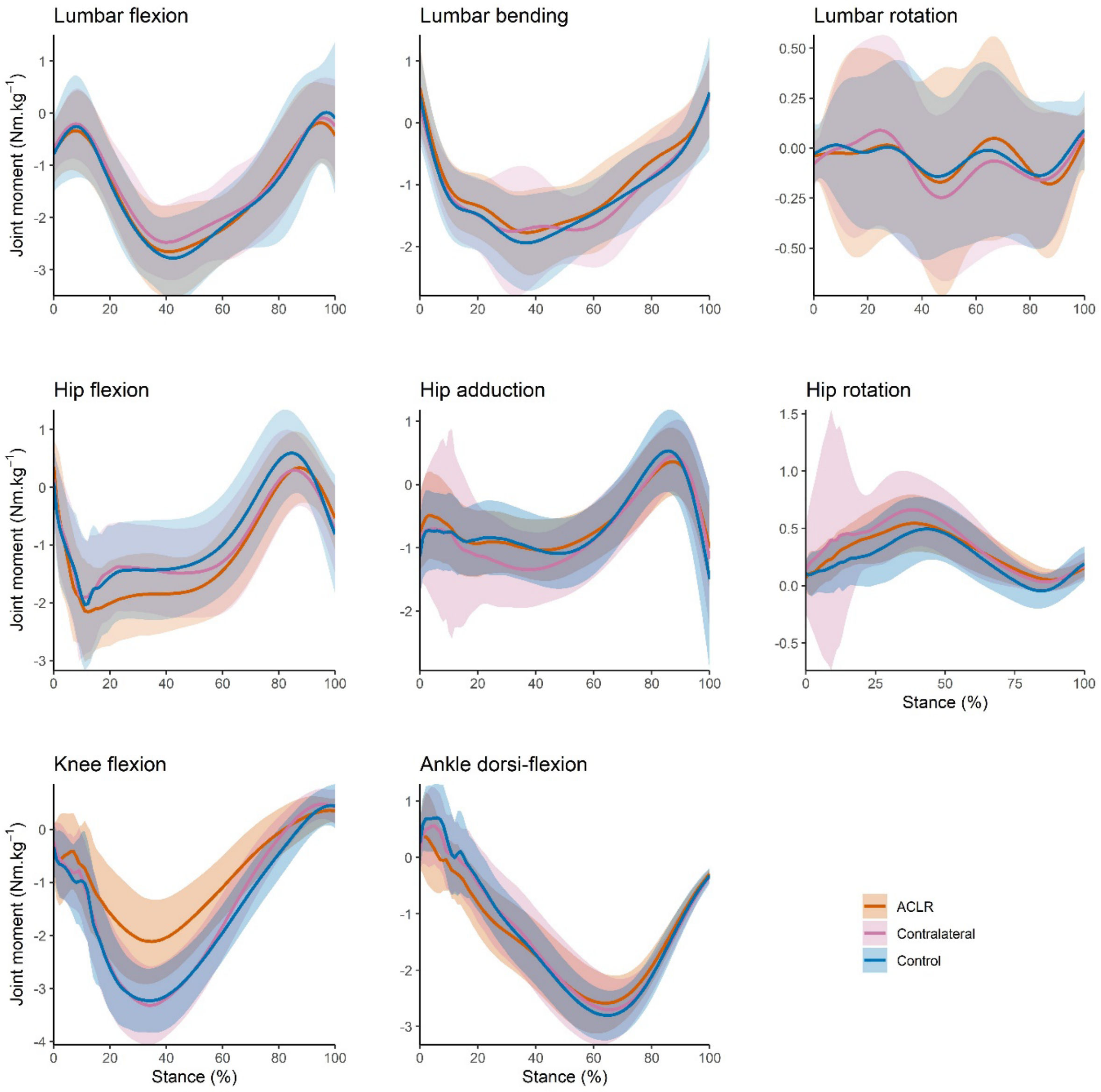

Figure A2. Mean (line) and SD (shaded area) lower limb joint moments during the stance phase of the anticipated sidestep cut between the ACLR (orange), Contralateral (pink), and Control (blue) limbs. Values are averaged across all participants and reported as Newton metres of torque normalised to body mass. *ACLR,* anterior cruciate ligament reconstruction; *Nm.kg*, Newton metres of torque normalised to body mass.

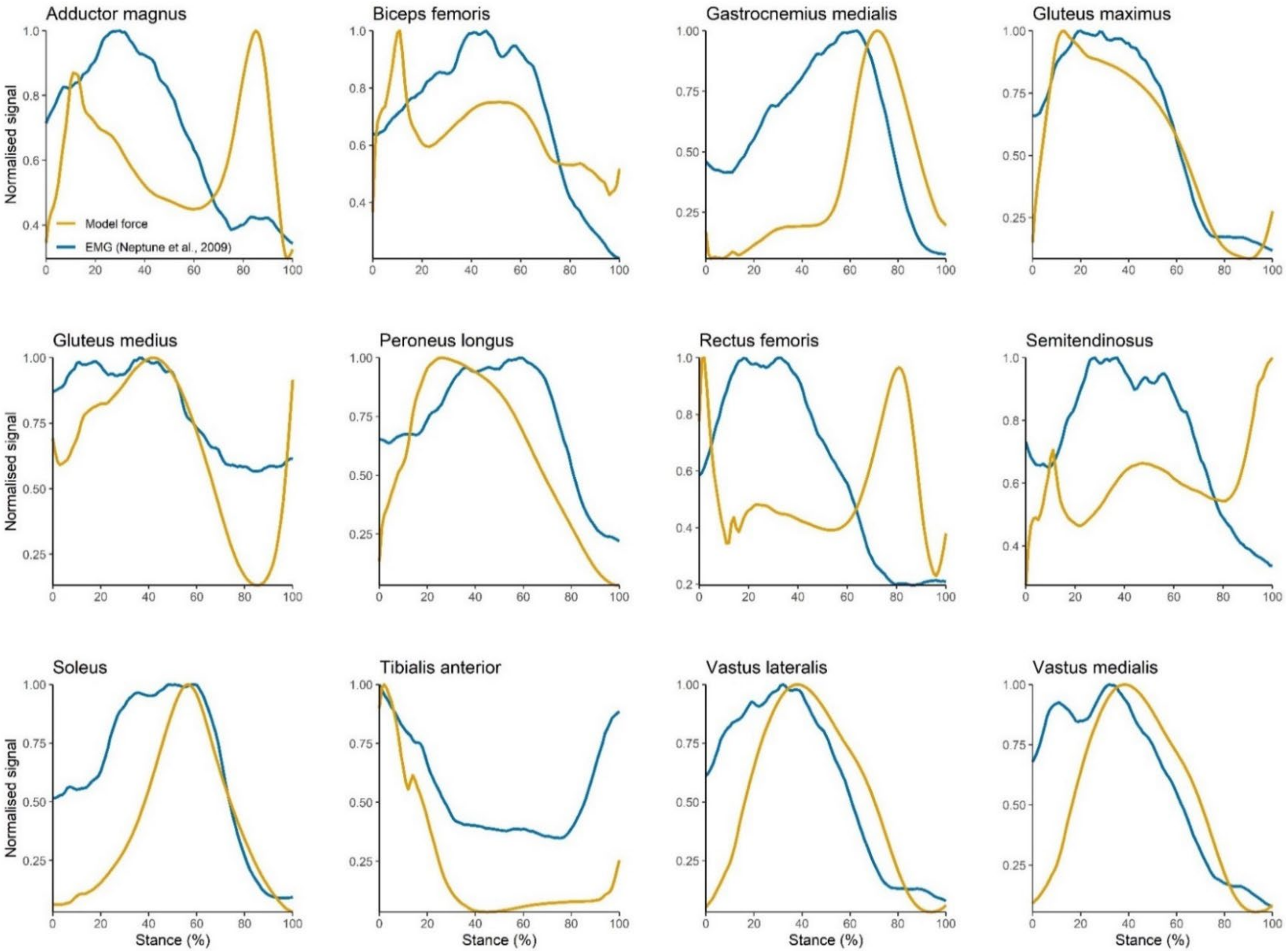

Figure A3. Comparison of predicted model force (yellow line) from the stance phase of the anticipated sidestep cut with the activations from electromyograph data from Neptune et al., 1999 (blue line)
